# Topological Analysis Reveals Multiple Pathways in Molecular Dynamics

**DOI:** 10.1021/acs.jctc.5c00819

**Published:** 2025-10-10

**Authors:** Luca Donati, Surahit Chewle, Dominik St Pierre, Vijay Natarajan, Marcus Weber

**Affiliations:** † 9166 Freie Universität Berlin, Department of Mathematics and Computer Science, Arnimallee 22, D-14195 Berlin, Germany; ‡ 39043 Zuse Institute Berlin, Takustr. 7, D-14195 Berlin, Germany; § Freie Universität Berlin, Department of Biology, Chemistry and Pharmacy, Arnimallee 22, D-14195 Berlin, Germany; ∥ 624471 Indian Institute of Science, Department of Computer Science and Automation, 560012 Bangalore, India

## Abstract

Molecular Dynamics simulations are indispensable tools for comprehending the dynamic behavior of biomolecules, yet extracting meaningful molecular pathways from these simulations remains challenging due to the vast amount of high dimensional data. In this work, we present Molecular Kinetics via Topology (MoKiTo), a novel approach that combines the ISOKANN algorithm to determine the membership function of a molecular system with a topological analysis tool inspired by the Mapper algorithm. Our strategy efficiently identifies and characterizes distinct molecular pathways, enabling the detection and visualization of critical conformational transitions and rare events. This method offers deeper insights into molecular mechanisms, facilitating the design of targeted interventions in drug discovery and protein engineering.

## Graphical abstract

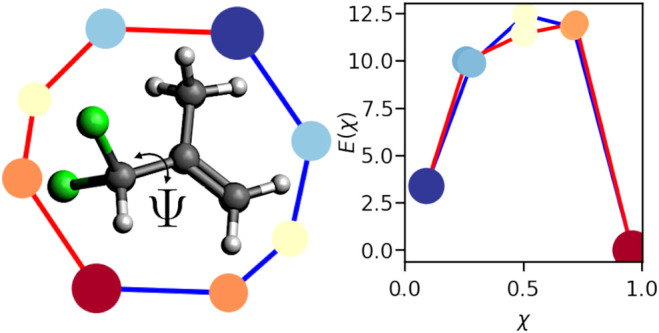

## I Introduction

The identification of reaction pathways in chemical processes such as protein folding, protein–ligand binding/unbinding and enzymatic reactions is fundamental to the development of novel drugs and clinical treatments.^1–4^ However, the long time scales of these processes, stemming from the ruggedness of the underlying energy landscapes, make it challenging to resolve these pathways via long Molecular Dynamics (MD) simulations. A widely adopted approach, which relies on the sampling of short MD simulations, is the construction of a Markov State Model (MSM),^5–8^ from which one can extract pathways linking the states of an initial macro-state to those of a target macro-state, by applying tools such as Transition Path Theory (TPT)^9–12^ or MSMPathfinder.^13^

MSM-based methods often reveal a large number of transition pathways, offering detailed mechanistic insight.^14^ However, these pathways can be difficult to visualize and interpret without clustering tools such as Path Lumping^15^ or Latent-space Path Clustering,^16^ which aim to simplify the network. Recently, new approaches that take advantage of machine learning techniques have been developed.^17–19^ Of particular interest is the method proposed in ref,^20^ where MD trajectories connecting the macro-states are generated via enhanced MD simulations^21^ and clustered using the Dynamic Time Warping (DTW) algorithm.^22^ This novel approach does not rely on MSMs and does not require dimensionality reduction, thereby resulting in a significant advancement over the state-of-the-art.

However, it requires a priori knowledge of the system to configure the enhanced sampling algorithm that generates the trajectories. Moreover, it relies on trajectory clustering, which is substantially more complex than clustering static points because it operates in a high-dimensional space where each object is a time series.

In this article, we propose a new but complementary framework called Molecular Kinetics via Topology (MoKiTo) for identifying pathway networks using tools derived from Topological Data Analysis (TDA). In MoKiTo, MD data can be generated either by conventional MD simulations or by enhanced sampling techniques, and are subsequently ordered and partitioned according to an ordering parameter χ: Γ → [0, 1], where Γ denotes the state space of the molecular system. The data are then connected through shared data points to form a graph which reveals macro-states, transition states, and pathways. Additionally, MoKiTo generates free energy profiles of individual pathways, facilitating their classification and the calculation of free energy differences and transition rates by Square Root Approximation.^23–25^

This method is inspired by the Mapper algorithm,^26^ a TDA tool designed to capture topological structure of large data sets by first ordering the data with respect to a parameter χ and then clustering points within intervals of χ. As an illustrative example, originally presented in ref 27, Figure 1 shows a point cloud sampled from a human hand clustered and connected in a graph using an ordering parameter that maps values from the wrist to the fingertips. Here, the wrist and fingertips play the role of metastable basins, and the single scalar χ is sufficient to reveal multiple pathways from a source to distinct targets. This example motivates our use of Mapper for MD data where multiple metastable basins and intricate networks of pathways are often present.

**Figure 1:**
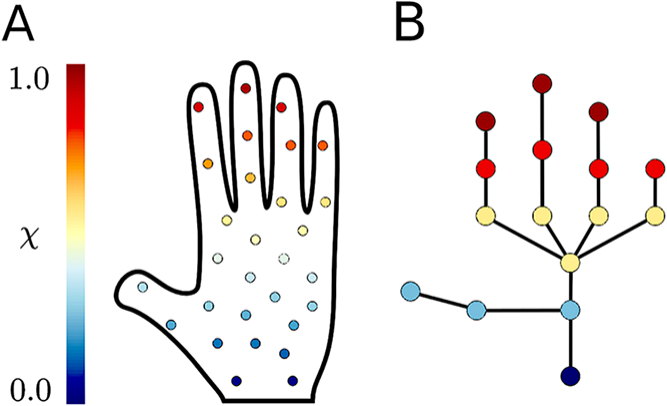
(A) Data points sampling a “hand” ordered according to a χ-function. (B) Graph realized with the mapper algorithm.

In the MD setting, χ acts as a reaction coordinate, then it should vary monotonically along the dominant slow mode of the system described by the second eigenfunction of the Koopman operator. In two-state scenarios, the committor is the optimal reaction coordinate^28–31^ and a natural choice for MoKiTo. Under reversibility and a clear spectral gap, the committor can indeed be approximated by the leading nontrivial Koopman eigenfunction. Alternatively, in this work we adopt as ordering parameter the so-called “membership function”, originally introduced in PCCA+.^32^ The membership function, hereafter the χ-function, is defined as an affine normalization of the leading nontrivial Koopman eigenfunction to [0, 1], thus describing the dominant slow mode of the system. Unlike the committor, it operates without having to specify source and target sets but is nevertheless consistent with the definition of natural reaction coordinate.^33^ Aside from technical details, which are clarified in the theory section, the significance of the χ-function is to describe the probability that a system’s conformational state belongs or does not belong to a macro-state.

To estimate the χ-function, we use ISOKANN,^34–36^ a data-driven method that trains an artificial neural network until convergence. The advantage of ISOKANN with respect to other methods such as PCCA+ ^32,37^ is that it does not need to discretize the space neither a low-dimensional featurization of the system. It operates directly on molecular coordinates and yields a smooth function that generalizes out of sample and can be evaluated on arbitrarily many configurations.

In MoKiTo, the choice of the ordering parameter remains flexible and arbitrary. Other valid options are the minimum energy path^38–40^ or the minimum action path.^41–44^ Alternatively, physical coordinates such as interatomic distances, bond angles, and dihedral angles can also serve as ordering parameters in this context. Likewise, the algorithm used to determine the ordering parameter is not fixed. Depending on the system and objectives, one may employ techniques such as Principal Component Analysis (PCA),^45,46^ Time-lagged Independent Component Analysis (TICA),^47,48^ Time-lagged Autoencoders (TAEs),^49^ diffusion maps,^50^ isometric feature mapping (ISOMAP),^51^ sketch-map,^52^ or others.

We applied MoKiTo to several molecular systems. Of particular interest is the fourth example, where we studied the villin headpiece subdomain,^53–56^ revealing both dominant and minor folding pathways. This detailed mapping of the folding landscape not only highlights the complexity of protein folding, but also emphasizes the importance of alternative routes that, despite being less frequent, contribute significantly to the overall dynamics of the protein.

## II Theoretical Background

Molecular systems often exhibit a broad separation of time scales, with fast thermal fluctuations that decay much faster than slow conformational rearrangements. A standard approach to characterizing such systems is the spectral analysis of the Koopman operator $K_{\tau }$ . This operator propagates bounded observables f:Γ→R , where Γ denotes the configuration space of the system, over a lag time τ, or equivalently through its infinitesimal generator L as

ft+τ(x)=exp(τL)ft(x)=Kτft(x)=E[ft(xt+τ)|xt=x] 3

where the last line expresses the action of the Koopman operator as a conditional expectation over trajectories. If the dynamics of the molecular system are governed by a confining potential energy function V(x):Γ→R , such that the equilibrium density

4 $$\pi \left(x\right)=\frac{1}{Z}e^{-\beta V\left(x\right)}$$

where *Z* is the normalizing constant, *β*=1/*(k*_B_ *T)* with temperature *T* and Boltzmann constant *k_B_*, is well-defined, then the operators $K_{\tau }$ and L are self-adjoint in the weighted space *L* ^2^(π), and the dynamics satisfies detailed-balance condition. Under these conditions, the eigenvalues and eigenfunctions of the two operators solve the eigenvalue problems

5 $$L\psi_{i}=\kappa_{i}\psi_{i}$$

6 $$K_{\tau }\psi_{i}=\lambda_{\tau ,i}\psi_{i}$$

with λ_τ,*i*_ = exp­(τκ_*i*_). The eigenvalues are sorted by decreasing magnitude, so that

7 $$\kappa_{0}=0>\kappa_{1}>\kappa_{2}\cdot \cdot \cdot$$

8 $$\lambda_{\tau ,0}=1>\lambda_{\tau ,1}>\lambda_{\tau ,2}\cdot \cdot \cdot >0$$

while the eigenfunctions form an orthonormal basis of *L* ^2^(π) with respect to the weighted scalar product ⟨*f, g*⟩_π_ = ∫*f*(*x*)*g*(*x*)­π­(*x*)­d*x*. The trivial eigenfunction ψ_0_ is constant, representing the equilibrium mode, whereas the leading nontrivial eigenfunction ψ_1_ is monotonic with a single node. It encodes the dominant slow relaxation process, orthogonal to equilibrium and provides a natural reaction coordinate for the system, as argued in ref 57, since it satisfies the following key properties: (i) it performs dimensionality reduction by mapping each state to a single real value; (ii) it is uniquely determined by the system’s dynamics, without requiring an a priori definition of macro-states; and (iii) it provides an optimal description of the dynamics by preserving both the Markovianity and the dominant implied time scales encoded in the Koopman operator. These properties make it convenient to introduce the normalized counterpart of ψ_1_, the so-called membership function (or χ-function), originally proposed in ref 32 as solution to the PCCA+ problem. The χ-function is defined as an affine rescaling of ψ_1_ to the interval [0, 1]

9 $$\chi \left(x\right)=a_{0}+a_{1}\psi_{1}\left(x\right)$$

where

10 $$\{\begin{array}{l} a_{0}=\frac{\max_{x}\psi_{1}\left(x\right)}{\max_{x}\psi_{1}\left(x\right)-\min_{x}\psi_{1}\left(x\right)}\\ a_{1}=-\frac{1}{\max_{x}\psi_{1}\left(x\right)-\min_{x}\psi_{1}\left(x\right)} \end{array}$$

By construction, the χ-function satisfies the same properties as ψ_1_, while offering a natural probabilistic interpretation: χ­(*x*) can be seen as the degree of membership of state *x* to one of the two metastable macro-states. Although its definition assumes the existence of two metastable states, its applicability is not restricted to two-state systems. When multiple metastable states are present, the leading nontrivial eigenfunction ψ_1_ still encodes the dominant slow relaxation process, distinguishing between two macroscopic sets of states separated by the slowest dynamical barrier. As a result, the χ-function represents an ideal candidate for the ordering parameter in the MoKiTo framework.

A valid alternative is the committor function *q*(*x*), solution of the backward Kolmogorov equation

11 Qq=0

with absorbing boundary conditions *q*(*x*) = 0 for *x* ∈ *A* and *q*(*x*) = 1 for *x* ∈ *B*, where *A* and *B* denote two disjoint sets representing the macro-states of the system. The committor is acknowledged as the optimal reaction coordinate for a two-state system with clearly defined boundary sets *A* and *B*,^28–31^ as its isosurfaces coincide with the transition surfaces of the dynamics of the system. Furthermore, the committor admits the spectral representation

q=∑ibiψi≈b0+b1ψ1 13

where *b*_*i*_ = ⟨*q*, ψ_*i*_⟩_π_ are the expansion coefficients of the committor in the eigenfunction basis and where the approximation holds for a two-state reversible system with a large spectral gap. In this case, the committor reduces to an affine transformation of the leading nontrivial eigenfunction, just as the membership function does. Therefore, both χ­(*x*) and *q*(*x*) induce the same ordering of configurations and essentially the same iso-surfaces. However, the χ-function can be obtained without any prior specification of sets *A* and *B*, making it more general than the committor and the preferred choice for the ordering parameter in MoKiTo. This advantage becomes particularly relevant in multistate landscapes, where suitable definitions of *A* and *B* may not exist, while the χ-function remains unambiguously defined.

## III Methods

MoKiTo is a framework that allows one to extract kinetic information from a set of short MD trajectories to construct graphs that highlight the dominant pathways between macro-states. This procedure, as outlined in Figure 2(A), can be summarized in three stages:

- The first stage focuses on exploration of the state space and propagation of dynamics by means of MD simulations.
- The second stage involves definition of an ordering parameter, the so-called χ-function, by means of the ISOKANN algorithm.
- The third stage uses a clustering algorithm to cluster the MD data filtered by the χ-function. Then the edges of the graph are assigned according to the overlap of neighborhoods.

**Figure 2:**
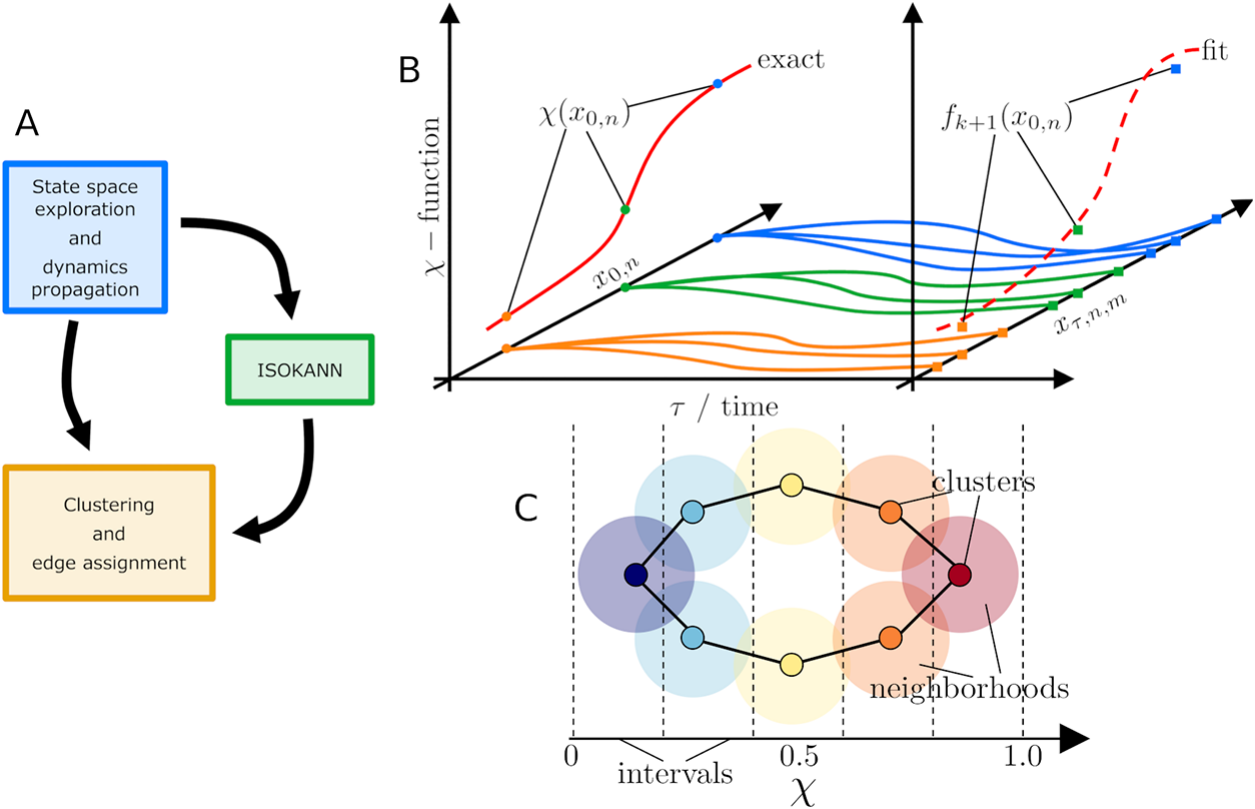
(A) MoKiTo workflow diagram. Constructing the MKM using a three-stage procedure. (B) ISOKANN scheme. Given *N* points *x_0,n_*, if the exact χ-function is not available, the procedure is as follows: (i) propagate *M* short trajectories starting from each *x_0,n_*, (ii) apply an arbitrary function *f_k_* to the final states *x_τ,n,m_* and estimate its average for each state *x_0,n_*, and (iii) apply the shift–scale function *S* as defined in eq 14. (C) Clustering and edge assignment scheme. The states representing the state space are first subdivided into intervals according to the χ-function. Then, the states of the same interval are clustered by CNN clustering algorithm and edges are found by overlapping the neighborhoods.

### III.I State Space Exploration and Dynamics Propagation

The objective of this stage is to identify *N* representative states of the state space Γ, which can be achieved through different methods depending on the system under investigation. As we will show in our numerical experiments, for systems characterized by low energy barriers, e.g., short chains of amino acids, conventional Molecular Dynamics or Monte Carlo simulations are the most convenient solutions. Instead, for bigger systems such as proteins, we recommend the use of enhanced techniques, such as Simulated Tempering MD (STMD) simulations,^58^ replica exchange MD,^59^ umbrella sampling,^60^ or metadynamics.^61–63^ Here, in the fourth example of chicken villin headpiece protein, we opted for STMD simulations, where the problem of getting stuck in local minima is overcome by dynamically adjusting the temperature during the simulation. STMD simulations are advantageous over the other proposed methods because they do not require a set of collective variables to be chosen a priori. However, states generated by STMD simulations require further minimization and equilibration to ensure they represent the equilibrium distribution of the canonical ensemble.

Once a set of *N* representative states has been built, it is necessary to propagate the dynamics. For this purpose, we perform *M* conventional MD simulations of length τ for each representative state. It is important to emphasize that, at this stage, the simulations must accurately reflect the true dynamics of the system under investigation. Consequently, enhanced sampling techniques that modify the potential energy surface or alter the system’s temperature are not applicable. From now on, we will use the notation *X*_0_ to denote the set of *N* initial states and *X*_τ_ to denote the set of *N* × *M* final states of MD trajectories of length τ.

### III.II ISOKANN

To compute the χ-function for a multidimensional system, we use ISOKANN,^34^ an iterative algorithm inspired by the Von Mises iteration method,^64^ that proceeds according to the update rule

14 $$f_{k+1}\left(x\right)=SK_{\tau }f_{k}\left(x\right)$$

where *S* is a linear transformation, $K_{\tau }$ is the Koopman operator and *f*_*k*_(*x*) is a real-valued function. The function *S*, based on the definition of χ-function in eq 7, is known as shift-scale function

15 $$SK_{\tau }f_{k}\left(x\right)=\frac{K_{\tau }f_{k}\left(x\right)-\min\left(K_{\tau }f_{k}\left(x\right)\right)}{\max\left(K_{\tau }f_{k}\left(x\right)\right)-\min\left(K_{\tau }f_{k}\left(x\right)\right)}$$

This transformation prevents the convergence of $K_{\tau }f_{k}\left(x\right)$ to the dominant trivial eigenfunction, ensures that the updated function remains within the interval [0, 1], and guides the convergence toward the desired χ-function

16 $$\lim_{k\to \infty }f_{k+1}\left(x\right)=\chi \left(x\right)$$

Since we do not know an analytical expression of $K_{\tau }$ , nor a matrix representation of it, we approximate the conditional expectation in eq 1 as

17 $$f_{k+1}\left(x_{0,n}\right)=S\frac{1}{M}\sum_{m=1}^{M}f_{k}\left(x_{\tau ,n,m}|x_{0}=x_{0,n}\right)$$

where *x*_τ,*n*,*m*_ ∈ *X*_τ_ is the final state of the *m*th trajectory started in *x*_0,*n*_ ∈ *X*_0_.

In this formulation, we assume an analytical expression for *f*_*k*_(*x*). However, the application of eq 14 yields only the scalar values *f*_*k*+1_(*x*_0,*n*_) at the sample points *x*_0,*n*_. Since we do not have an explicit analytical form for *f*_*k*+1_(*x*), the question arises: how can we apply the Koopman iteration at the next step? To address this, we seek an analytical function that best fits the *N* scalar values *f*_*k*+1_(*x*_0,*n*_)_*n*=1_^*N*^ using regression techniques. For low-dimensional systems, methods such as spline interpolation or radial basis functions are often preferable due to their simplicity and low number of trainable parameters. In contrast, for high-dimensional systems, Feedforward Neural Networks (FNNs) are recommended due to their superior computational power and scalability. In the examples presented in this manuscript, we employed an FNN, whose training procedure is described in the SI. Figure 2(B) summarizes the ISOKANN procedure.

### III.III Clustering and Edge Assignment

We subdivide the χ-function into *N*_*I*_ disjoint intervals containing states with similar χ-value. The number of intervals into which the χ-function is subdivided is arbitrary, but ideally there should be a sufficient number of states representing similar macroscopic behavior, i.e., macro-states and transition states. This partial clustering based on the χ-function is useful to reduce the complexity of the data while preserving important properties such as Markovianity.^65^

Once the intervals of the χ-function have been defined, we cluster states in state space Γ. Several algorithms could be used. Here, we chose the Common Nearest Neighbor (CNN) clustering algorithm,^66^ an unsupervised clustering algorithm that uses local density information to identify clusters of data points without prior knowledge of the number of clusters. CNN clustering tends to work well with nonlinearly separable data and has already been shown to be suitable for the study of molecular systems.^67,68^ The key assumption of this algorithm is that two states are more likely to belong to the same cluster if they share a significant number of neighbors. The algorithm is then controlled by two key parameters: the radius of the neighborhood ε and the number of nearest neighbors θ. Two states are considered neighbors if they are less than a distance ε apart from each other

18 $$x_{i}\;\mathrm{and}\;x_{j}\;\mathrm{are}\;\mathrm{neighbors}\;\mathrm{if}\;\left|x_{i}-x_{j}\right|<\varepsilon$$

Then, for every pair of states, the intersection of their respective nearest neighbor sets is determined: if *x*_*i*_ and *x*_*j*_ share at least θ neighbors, they belong to the same cluster. The algorithm determines *K* clusters Ω_1_, Ω_2_, ···, Ω_*K*_, where *K* is an output parameter, however, like many density-based methods, its performance is sensitive to the choice of θ and ε. Following the indications in refs 66,67 , the parameters should be chosen on the basis of the histogram of pairwise Root Mean Square Distances (RMSDs) between the states in the data set: ε should be set to a value slightly smaller than the first maximum of this histogram, while θ is varied until adequated sized clusters are found.

The last step in the construction of the graph is the assignment of edges, i.e., finding the connections between pairs of clusters Ω_*i*_ and Ω_*j*_ ∀*i*, *j* = 1, 2, ···, *K*. For this purpose, we assume that a transition in an infinitesimal time span can only take place between similar conformations, both macroscopically and microscopically. Thus, to determine edges, we look for clusters belonging to consecutive intervals that have common states in their neighborhood. Indeed, a transition between clusters in consecutive intervals indicates a transition between states with similar macroscopic properties and clusters that share states in their neighborhood, ensuring that the transition occurs between states with similar conformational structure. In practice, the procedure begins by aligning the states within a cluster to minimize the RMSD and computing the average structure of the cluster. Then, the neighborhood of the cluster, whose size is determined by a threshold *r*_*n*_, is identified by calculating the RMSD between the average structure and all the states in the *X*_0_ data set. The procedure to determine the clusters and the edges is schematized in Figure 2(C).

The resulting graph, referred to as the Molecular Kinetics Map (MKM), consists of clusters ordered according to the values of the χ-function. The initial cluster Ω_0_ (with χ ≈ 0) and the final cluster Ω_*K*_ (with χ ≈ 1) represent the dominant macro-states of the system; typically reactants and products in a chemical reaction, or, in the context of biomolecules, the unfolded–folded states of a protein, bound-unbound conformations, and so on. The MKM can then be used to identify the principal pathways connecting the macro-states. We define an arbitrary pathway χ^*p*^ connecting the initial cluster Ω_1_ to the final cluster Ω_*K*_ as a sequence of clusters along the reaction coordinate χ as

19 $$\chi^{p}=\left\{\Omega_{p_{1}},\Omega_{p_{2}},\cdot \cdot \cdot ,\Omega_{p_{L}}\right\}$$

where *L* is the length of the pathway, Ω_*p*_1__ = Ω_1_ and Ω_*p*_*L*__ = Ω_*K*_ are the initial and final clusters of the MKM respectively, and each Ω_*p*_*j*__ = Ω_2_, Ω_3_, ···, Ω_*K*–1_.

### III.IV Free Energy Profiles and Calculation of Transition Rates

We assume that the *N* initial states *x*_0_, *n* ∈ *X*_0_ are distributed according to the canonical equilibrium distribution defined in eq 2. Then, given a pathway χ^*p*^ as defined in eq 16, the probability distribution projected onto the clusters Ω_*p*_*i*__ of the pathway is defined as

20 $$\pi \left(\Omega_{p_{i}}\right)=\frac{\int_{\Gamma }\delta \left[\Omega_{p_{i}}\left(x\right)-\Omega_{p_{i}}\right]\exp\left(-\beta V\left(x\right)\right)dx}{\int_{\Gamma }\exp\left(-\beta V\left(x\right)\right)dx}$$

where δ is the Dirac δ-function. Equation 18 describes the Boltzmann weight of each cluster of the pathway and it is associated with the free energy

21 $$E\left(\Omega_{p_{i}}\right)=-\frac{1}{\beta }\log\pi \left(\Omega_{p_{i}}\right)$$

Plotting the free energy along each pathway as a function of the reaction coordinate χ provides a natural way to classify pathways according to their thermodynamic likelihood; i.e., pathways traversing lower free energy barriers are more probable. Applying the Square Root Approximation method (SqRA)^23–25^ we approximate the transition rate between adjacent clusters of the same pathway as

22 $$k_{p_{i},p_{i+1}}\propto \sqrt{\frac{\pi_{p_{i+1}}}{\pi_{p_{i}}}}$$

and we calculate the effective transition rate from the initial to the final state of a pathway as

23 $$k^{p}={\left(\sum_{i=1}^{L-1}\frac{1}{k_{p_{i},p_{i+1}}}\right)}^{-1}$$

which corresponds to the harmonic mean of the inverse transition rates along the pathway.

## IV Results

### IV.I Two-Dimensional System

As an illustrative example, we considered a two-dimensional system governed by overdamped Langevin dynamics and defined by the potential energy function

24 $$V\left(x,y\right)=10{\left(x^{2}-1\right)}^{2}+5\mathrm{xy}+10{\left(y^{2}-1\right)}^{2}+2.2x$$

illustrated in Figure 3(A). The potential is characterized by 4 local minima of different height: the deepest is corner *C*_1_ = (−1, 1), followed by *C*_4_ = (1, −1), *C*_3_ = (−1, −1) and *C*_2_ = (1, 1), in order from lowest to highest. To give physical meaning to the problem, we assumed that the potential has energy units kJ mol^–1^, and that generates forces −∇_*x*_ *V* and −∇_*y*_ *V* on a fictitious particle of mass *m* = 1 amu that moves on a flat surface. We also assumed standard thermodynamic parameters: the temperature of the system was *T* = 300 K with molar Boltzmann constant *k*_B_ = 8.314 × 10^–3^ kJ K^–1^ mol^–1^. This choice of the parameters makes sure metastability, indeed the system’s thermal energy β^–1^ = *k*_B_ *T* = 2.49 kJ mol^–1^ is significantly smaller than the height of the barriers along *x* and *y*. The interaction of the particle with the environment is modeled via a friction coefficient γ = 1 ps^–1^ and a diffusion constant *D* = *k*_B_ *T*/*m*γ = 2.49 nm^2^ ps^–1^ in each direction.

**Figure 3:**
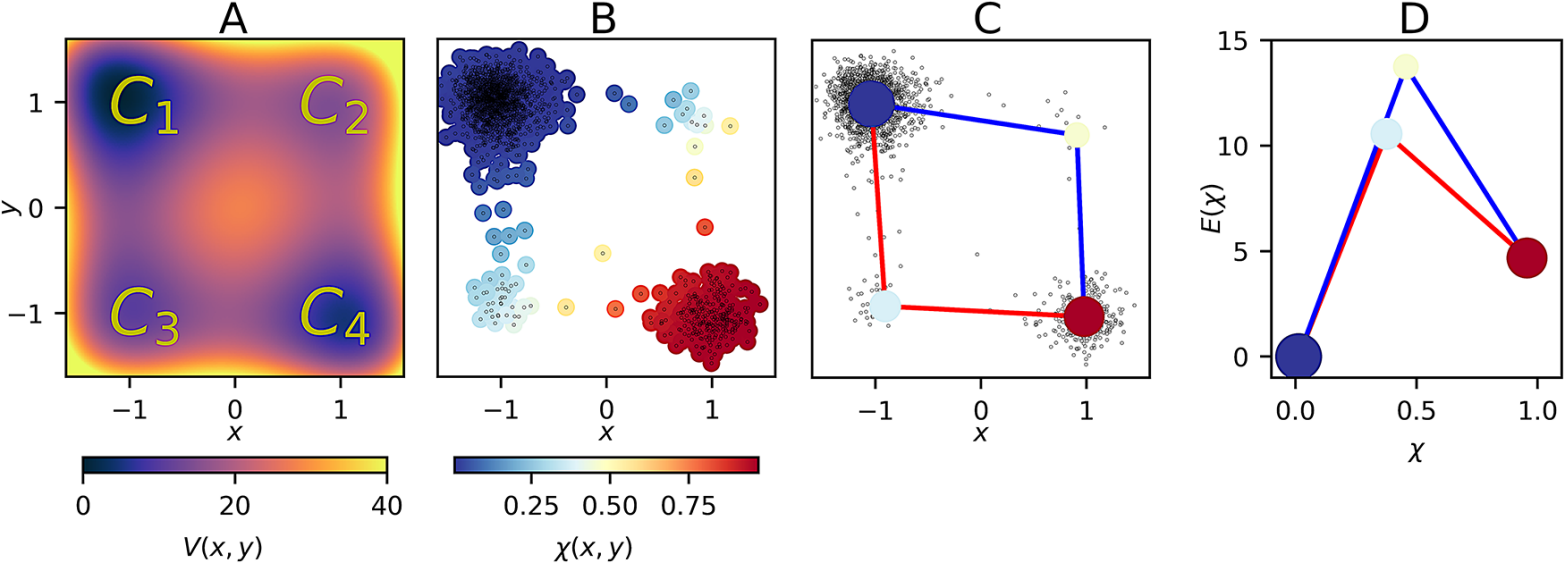
Results of the two-dimensional system. (A) Potential energy function; (B) initial states extracted from a trajectory and colored according to the membership function χ­(*x*, *y*). (C) MKM projected onto the Cartesian space. The black dots represent the initial states; (D) Free energy profiles of the pathways.

#### IV.I.I State Space Exploration and Dynamics Propagation

We solved the overdamped Langevin dynamics equations of motion

25 $$\{\begin{array}{l} dx_{t}=-\beta D\nabla_{x}V\left(x_{t},y_{t}\right)dt+\sqrt{2D}dW_{x}\\ dy_{t}=-\beta D\nabla_{y}V\left(x_{t},y_{t}\right)dt+\sqrt{2D}dW_{y} \end{array}$$

where *W*_*x*_ and *W*_*y*_ are two independent and uncorrelated Wiener processes, applying the Euler-Maruyama scheme^69^ with a time step of Δ*t* = 0.001 ps.

First, we generated a sufficiently long trajectory of 1 × 10^7^ timesteps which covers the relevant regions of the potential. Then, we extracted 4000 initial states equally spaced from the trajectory, i.e., one each 1000 timesteps, and carried out 10 short trajectories of 10 timesteps from each initial state.

As suggested in the Methods section, we organized the data into two arrays: *X*_0_ of shape (4000,2) containing the coordinates of the initial states, and *X*_τ_ of shape (4000,10,2) containing the coordinates of the final states of the short trajectories.

#### IV.I.II ISOKANN

To construct the χ-function, we applied ISOKANN. For regression, we used an FNN with three layers and the sigmoid function as activation function. The FNN was implemented using PyTorch,^70^ the input layer had 2 nodes, one for each coordinate of the system, the hidden layer had 128 nodes and the output layer had 1 node corresponding to the χ-value. The optimization of the FNN parameters was performed using the Stochastic Gradient Descent (SGD) algorithm^71^ and minimizing the mean squared error (squared $l^{2}$ -norm). At each ISOKANN iteration, we trained for 15 epochs, iterating over randomly generated batches of size 100, with an initial learning rate of 0.001. This choice of hyperparameters is the result of a random search and leads to a convergence of the χ-function in 4 iterations (SI, Figure S1). To validate the model, we monitored training and validation losses and observed that they decrease in parallel without an increase in the gap or a rebound in validation loss, indicating no evidence of overfitting.

The χ-function, evaluated at each initial point *X*_0_, is illustrated in Figure 3(B). The corners *C*_1_ and *C*_4_, colored by blue (χ ≈ 0) and red (χ ≈ 1) respectively, are the two main macro-states, i.e., the regions of state space most visited by the particle. Corners *C*_3_ and *C*_2_ are colored with a gradient of colors blue-yellow (χ ≈ 0.5), and can be interpreted as transition state states.

#### IV.I.III MKM Construction

To construct the MKM representing the macro-states and the main pathways of the dynamics, we proceeded in two steps: first we grouped the states by dividing the χ-function into 3 regular intervals and finding for each interval 1703, 32, and 263 states (*x*, *y*) respectively, then we applied the CNN clustering algorithm to group the states into smaller clusters having similar χ-value. For the CNN clustering algorithm, we chose as radius of the neighborhood ε = 1.0 and as number of nearest neighbors θ = 5, finding *N*_*c*_ = 4 clusters. To connect the clusters, we searched for areas of overlap between neighborhoods of clusters belonging to consecutive intervals, using as a threshold the Euclidean distance *r*_*n*_ = 0.6. The MKM is shown in Figure 3(C).

#### IV.I.IV Observations

The potential energy function has four macro-states and by means of the χ-function we identify two macroscopic regions at corners *C*_1_ and *C*_4_, and one transition region that includes both corners *C*_2_ and *C*_3_. There are two main pathways from corner *C*_1_ to corner *C*_4_ and vice versa. In Figure 3(D), we show the free energy profiles as defined in eq 19. Since the blue and red clusters are the largest, their energy levels are low, the yellow clusters, instead, are higher and correspond to transition states. From this graph, we also deduce that the red path (*C*_1_ – *C*_3_ – *C*_4_) is the most likely path, as the system needs less energy to visit corner *C*_3_ than corner *C*_2_. By SqRA (eqs 20 and 21), we estimated the effective transition rates of the two paths: *k*_+_^*r*^ = 0.07 and *k*_+_^*b*^ = 0.15 for the red and blue path, respectively. Thus, the reaction along the blue path is about 2.14 times faster than the red one. We estimated also the transition rates for the reverse reactions: *k*_–_^*r*^ = 0.18 and *k*_–_^*b*^ = 0.40 respectively.

### IV.II 33-Dichloroisobutene

As the first molecular system example, we studied 3,3-Dichloroisobutene (C_4_H_6_Cl_2_), a dichloro derivative of isobutene, represented in Figure 4(A). The compound has 12 atoms, for a total of 36 dimensions, and the rotation around the torsion angle Ψ (C_3_–C_2_-C_4_–Cl_1_) is known to be the slowest process of the system. Thus, we used the torsion angle Ψ as relevant coordinate to visualize the results.

**Figure 4:**
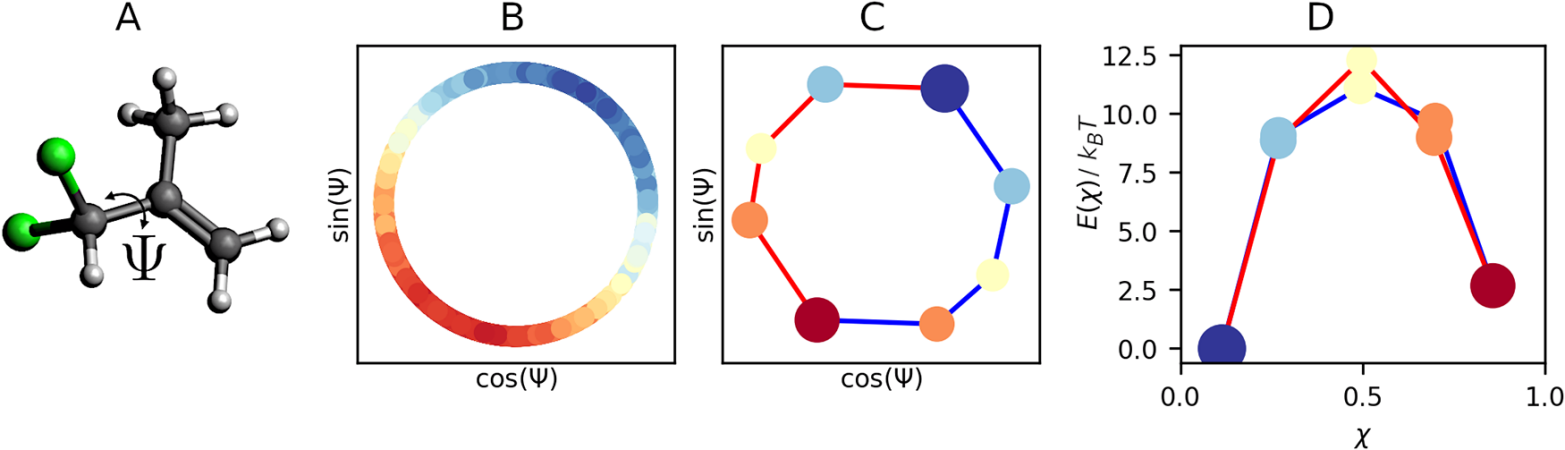
Results of 33-Dichloroisobutene molecule. (A) 33-Dichloroisobutene molecule; (B) χ-function projected onto the main torsion angle; (C) MKM projected onto the main torsion angle; (D) Free energy profiles of the pathways.

#### IV.II.I State Space Exploration and Dynamics Propagation

We performed MD simulations using the package OpenMM^72^ with the Generalized Amber Force Field.^73^ To simulate an explicit solvent, we used the TIP3P-FB water model^74^ with a padding distance of 1.4 nm which generates a box of 682 water molecules for 33-Dichloroisobutene. We assumed Langevin dynamics and applied the Langevin leapfrog integrator^75^ with γ = 1.0 ps^–1^ as friction coefficient, and Δ*t* = 2 fs as integrator time step. Nonbonded interactions between atoms, such as Coulomb forces and Lennard–Jones forces, were calculated by Particle-Mesh Ewald (PME) method^76^ and interactions between atoms more than 1 nm apart were truncated. The lengths of all bonds involving a hydrogen atom have been constrained. Before doing the first simulation, we brought the system to a local energy minimum, then we equilibrated the system with a 20 ps simulation to obtain a state belonging to the NVT ensemble, with temperature equal to 300 ± 6 K.

From a trajectory of 40 × 10^6^ timesteps, corresponding to 80 ns, we extracted 4000 states *x*_0_ = {*r*_0_, *v*_0_} (positions and velocities of each atom including the solvent) every 8 ps. Then the states *x*_0_ have been used as initial states for 10 new short trajectories of length 0.02 ps.

#### IV.II.II ISOKANN

The procedure for constructing the χ-function via ISOKANN was the same as in the previous example. However, instead of providing the Cartesian coordinates of the atoms, we used the pairwise distances between all the atoms of the system (without the water). This increases the number of dimensions of the χ-function to 12·(12–1)/2 = 66, but ensures that χ is invariant with respect to translations and rotations. As a model to approximate the χ-function, we used an FNN with 4 layers (2, 66, 33, 1 nodes), however, as activation function we used Leaky ReLU which performs better than sigmoid in high-dimensionality regression tasks. We set the initial learning rate for the SGD algorithm to 0.01 and applied a weight decay of 0.01 for regularization. The model converged after 279 iterations, achieving training and validation losses smaller than 10^–3^; no train–validation divergence or validation-loss rebound was observed, and χ was stable over ISOKANN iterations (SI, Figure S2).

The χ-function, evaluated in each initial point *x*_0_, is reported in Figure 4(B). For ease of reading, we have projected the χ-function onto the unit circle, i.e., the values cos­(Ψ) and sin­(Ψ), where Ψ is the torsion angle. We clearly distinguish macro-states colored with red and blue, and the transition states colored with yellow. The correlation between χ-function and torsion angle Ψ is equal to 0.9, confirming that the latter is a good choice as a relevant coordinate to describe the slowest process of the system.

#### IV.II.III Clustering and Edge Assignment

To construct the MKM, we discretized the χ-function into 5 equal intervals, then applied the CNN clustering algorithm. Clustering was done by precomputing the Root Means Square Distance (RMSD) matrix. Then, after analyzing the distribution of RMSDs, we identified the CNN clustering parameters: ε = 0.09, 0.08, 0.09, 0.07, 0.09, and θ = 5 (for each interval). With this setting, we obtained 8 clusters, 2 for macro-states and 6 for transition states. To find edges, we used as threshold for the neighborhoods *r*_*n*_ = 0.05. The MKM projected onto the unit circle of the angle Ψ is shown in Figure 4(C).

#### IV.II.IV Observations

We observe two main clusters: the red one (Ψ ≈ π/4) corresponds to a conformational state where both chlorines are staggered with the methylene group; the blue one (Ψ ≈ 3π/4) corresponds to a conformational state where both chlorines staggered with the methyl group. The two macro-states are connected by two pathways that correspond to the rotations of the torsion angle Ψ. The free energy profiles, illustrated in Figure 4(D), also reveal the energy barriers of the paths. Since the system is perfectly symmetrical with respect to the rotation of the Ψ angle, the two pathways overlap. In other words, no direction is preferred, and the system can rotate clockwise or counterclockwise with the same probability. However, we observe that there is a higher barrier between the red cluster and subsequent orange clusters than between the blue cluster and subsequent light blue clusters. Then the configurational states belonging to the red cluster are the most stable of the system. The effective transition rates of the two pathways are *k*_+_^*r*^ = 0.11 and *k*_+_^*b*^ = 0.11 for the red and blue path respectively; while for the reverse reactions are *k*_–_^*r*^ = 0.16 and *k*_–_^*b*^ = 0.16 respectively. This confirms the symmetry of the system and the equivalence of the two pathways.

### IV.III Hexapeptide VGVAPG

The VGVAPG is an elastin-derived hexapeptide,^77^ already used to test methods for MD simulations.^78,79^ The peptide has 73 atoms, corresponding to 219 dimensions in the Euclidean space. As relevant coordinate, denoted by *r*_*ee*_ in the figures, we used the Euclidean distance between the nitrogen atom of the N-terminus and the carboxyl-carbon of the C-terminus (Figure 5(A)).

**Figure 5:**
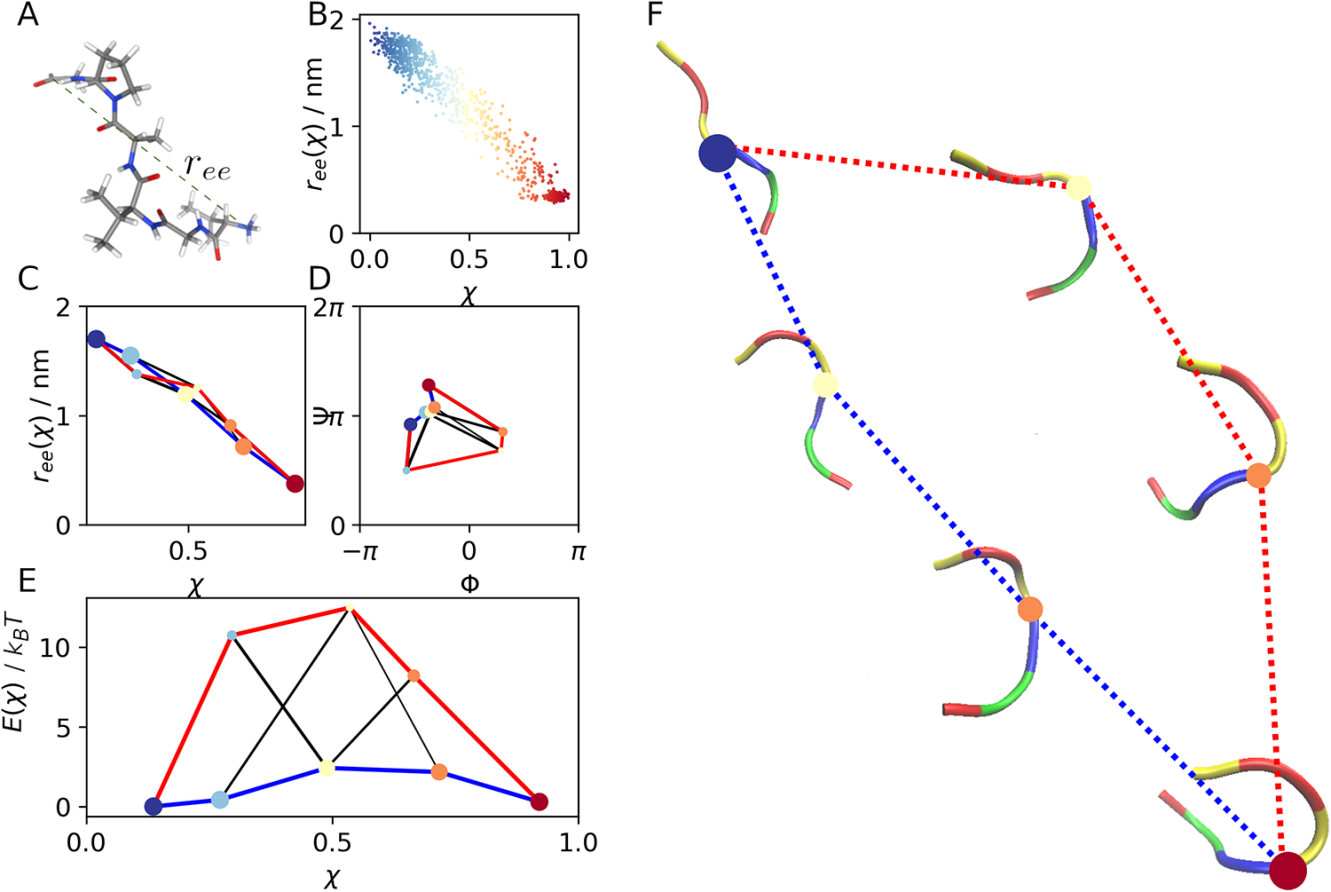
Results of VGVAPG hexapeptide. (A) VGVAPG molecule; (B) χ-function projected onto the end-to-end distance of the molecule; (C) MKM projected onto the end-to-end distance of the molecule. (D) MKM projected onto the Ramachandran plot of the second residue (Glycine 1); (E) Free energy profiles of the pathways. (F) MKM with representative structures. The colors of the molecular structures represent the residues: Val (yellow), Gly (red), Pro (blue) and Ala (green).

#### IV.III.I State Space Exploration and Dynamics Propagation

The MD simulations were carried out with the same settings as for 33-Dichloroisobutene, but the water box was increased to 782 water molecules and the force field was the AMBER ff-14sb.^80^ The length of the first simulation was 500 × 10^6^ timesteps, corresponding to 1 μs, from which, we extracted 1000 initial states for the short trajectories. For each initial state, we produced 10 short trajectories of 1000 timesteps, corresponding to 2 ps.

#### IV.III.II ISOKANN

We performed a random search to find the best hyper-parameters of the neural network and determined as optimal parameters, 1752 nodes in the hidden layer, 0.001 as initial learning rate and 0.005 as weight decay. The ISOKANN algorithm was performed for 57 iterations, training and validation losses stabilized around 4 × 10^–4^ with no train–validation divergence, and χ remained stable (SI, Figure S3). The χ-function is plotted in Figure 5(B). We observe a large macro-state, corresponding to χ ≈ 0.0 (blue), which includes configurations whose relevant coordinate *r*_*ee*_ ranges from 0.7 to 2 nm; the transition states, with χ ≈ 0.5 (yellow), range from 0.35 to 1.7 nm and the macro-state corresponding to χ ≈ 1.0 (red), includes configurations whose relevant coordinate ranges from 0.25 to 0.35 nm. The correlation between χ and *r*_*ee*_ is 0.98.

#### IV.III.III MKM Construction

We divided the χ-function into 5 equal intervals between 0 and 1. Then, by analyzing the distribution of RMSDs, we determined the parameters for the CNN clustering: ε = 0.3, 0.23, 0.15, 0.17, 0.3 and θ = 5 for each interval. Thus, we found 1, 2, 2, 2, and 1 cluster for each interval, respectively. The MKM, obtained with *r*_*n*_ = 0.3, is reported in Figure 5, where we propose different representations: in Figure 5(C), we show the complete MKM projected onto the end-to-end distance of the peptide; in Figure 5(D) we show the MKM projected onto the Ramachandran plot of the first Glycine (G1) of the peptide; in Figure 5(E), we show the free energy profiles; in Figure 5(F), we show the main representative structures of the backbone of the peptide (omitting the less relevant clusters of the MKM)

#### IV.III.IV Observations

The blue cluster (χ ≈ 0) comprises completely open structures with *r*_*ee*_ > 1.5 nm, while the red cluster (χ ≈ 1) represents closed structures with *r*_*ee*_ ≈ 0.3 nm. Since distance *r*_*ee*_ is highly correlated with χ, we cannot distinguish multiple paths from Figure 5(C). Thus, to better characterize the dynamics and describe the opening-closing mechanism of the peptide, we analyzed the Ramachadran plot of each residue. Here, in Figure 5(D), we report the Ramachadran plot of the second residue (the first Glycine in the chain VGVAPG), as it shows the most interesting dynamics. First, we observe that most of the clusters, in particular the clusters belonging to the blue path, are located in quadrant II and III where Φ < 0. We therefore deduce that the closure of the hexapeptide along the blue pathway does not lead to a significant rotation of the Glycine torsion angles. However, one orange cluster (χ ≈ 0.7) is located in quadrant IV (Φ ≈ 0.95), indicating that the red pathway involves a wide rotation of the Φ torsion angle of the Glycine: first about 120 degrees anticlockwise, then again about 110 degrees clockwise. The effective transition rates of the two pathways are *k*_+_^*r*^ = 0.09 and *k*_+_^*b*^ = 0.23 for the red and blue path respectively; while for the reverse reactions are *k*_–_^*r*^ = 0.12 and *k*_–_^*b*^ = 0.24 respectively. We conclude that the red pathway is more energy-intensive and, consequently, less probable.

### IV.IV Villin Headpiece Subdomain

As a last example, to demonstrate the applicability of our approach to large systems, we studied the villin headpiece subdomain^53–56^ which is one of the most studied protein for understanding protein folding. Villin consists of 35 residues (582 atoms), and in its folded structure, it forms 3 α-helices as shown in Figure 6(A): residues 4–8 form helix *H*_1_ (green), residues 15–18 form helix *H*_2_ (yellow), residues 23–32 form helix *H*_3_ (orange). To have a 3-dimensional representation of the molecular system, we used the RMSD of the helices $A$, $B$ and $C$ with respect to the corresponding segments of the X-ray crystal structure solved at pH 6.7 deposited in the RCSB protein data bank repository (PDB ID: 1YRF^81^) as done in ref 55. Thus, a structure with low RMSD values corresponds to a folded structure, a structure with high RMSD values is an unfolded structure, and partially folded structures correspond to a situation where only the RMSD of one segment has low values.

**Figure 6:**
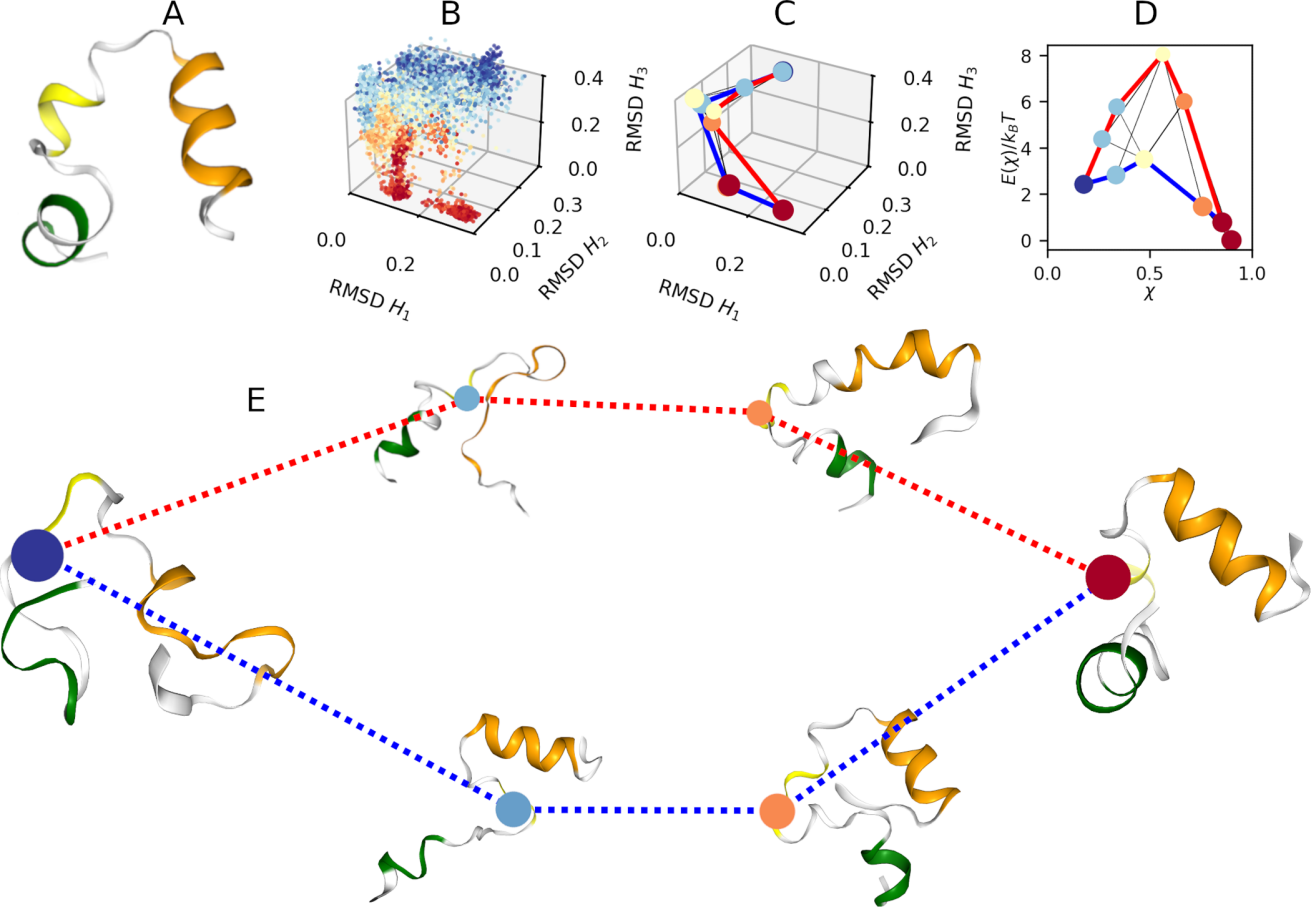
Results of villin headpiece subdomain. (A) The X-ray crystal structure of villin headpiece solved at pH 6.7, colors identify the helix *H*_1_ (green), *H*_2_ (yellow) and *H*_3_ (orange) respectively; (B) χ-function projected onto the RMSD of helices; (C) MKM projected onto the RMSD of helices; (D) Free energy profiles of the pathways; (E) MKM with representative structures of the molecule.

#### IV.IV.I State Space Exploration and Dynamics Propagation

The folding time scale of the villin protein is 2.8 μs, but a complete exploration of the space of states requires a conventional MD simulation of more than 2.8 μs.^56^ Alternatively, we carried out STMD simulations utilizing the dedicated OpenMM module for exploring the state space and selecting representative structures. First, we prepared a complete extended structure with PyMol.^82^ Then, we minimized the structure and equilibrated the system for 20 ps reaching a partially folded structure. At this point, we carried out 6 independent replicas of 1 μs with temperatures ranging from 273 to 500 K. All the other parameters and options were as in the previous examples, the box contained 2713 water molecules. From each replica, we extracted 1000 structures, for a total of 6000 structures, which constitute the set *X*_0_ of initial states. However, since temperature was a dynamic variable, we further equilibrated these structures for 100 ps to have a sample representing the Canonical Ensemble at *T* = 300 K. Afterward, we ran 10 short MD simulations of 1000 timesteps (0.2 ps) for each initial state.

#### IV.IV.II ISOKANN

The ISOKANN algorithm was applied as before, but we changed the input coordinates. Indeed, since the system has 582 atoms, the number of pairwise distances is 582·(582–1)/2 = 169071. Modern neural networks are able to handle this dimensionality, however, as a matter of efficiency and to show the versatility of the method, we preferred to reduce the dimensionality by using the internal coordinates (bonds, angles and torsion angles) of the backbone (140 atoms). In this way, we reduced the number of dimensions to 1716. We used a neural network with four layers (1716, 858, 429, 1), the initial learning rate of the SGD algorithm was 0.01 and the weight decay 0.005. Convergence of the training occurred in 95 iterations with a training and validation loss in the order of 10^–3^ (SI, Figure S4). Convergence was reached within 95 iterations, training and validation losses settled on the order of 10^–3^, decreasing in parallel with no divergence, and χ remained stable across iterations (SI, Figure S4).

In Figure 6(B), we report the χ-function projected onto the two collective variables. The bottom left corner (small values of RMSDs), contains folded structures with χ ≈ 1 (red). As we move away from the corner, we observe transition structures with χ ≈ 0.5 (yellow) up to a large area containing unfolded structures with χ ≈ 0 (blue).

#### IV.IV.III MKM Construction

We divided the χ-function into 5 equal intervals and determined the optimal parameters ε = 0.9, 0.5, 0.3, 0.5, 0.5 and θ = 10, 60, 50, 150, 20 for the CNN clustering algorithm and *r*_*n*_ = 0.6 to determine the edges. Thus, we found 10 clusters, two clusters correspond to fully folded and unfolded structures, while the others contain partially folded structures. The MKM is shown in Figure 6 in several representations: Figure 6(C) shows the projection onto the RMSDs of the helices; Figure 6(D) shows the free energy profiles as a function of the χ-values. (E) shows the pathways between the most relevant representative structures of the protein (omitting the less relevant clusters of the MKM).

#### IV.IV.IV Observations

We identify multiple folding pathways in the transition from unfolded to folded state, each with a distinct energetic profile. We have highlighted in blue the pathway that requires less energy, and in red the one that requires more, then the blue pathway is the most likely folding route. This pathway corresponds to a scenario in which helix *H*_3_ reaches its folded state more rapidly than the other two helices. Conversely, the red pathway exhibits a process in which helix *H*_1_ stabilizes prior to helices *H*_2_ and *H*_3_. In both cases, the formation of helix *H*_2_ is the slowest process and it starts at χ > 0.5. The effective transition rates of the two pathways are *k*_+_^*r*^ = 0.21 and *k*_+_^*b*^ = 0.26 for the red and blue pathway respectively; while for the reverse reactions are *k*_–_^*r*^ = 0.18 and *k*_–_^*b*^ = 0.22 respectively.

### IV.V Sensitivity Analysis to χ-Discretization and CNN Parameters

To assess the robustness of MoKiTo, we quantified how the MKMs depend on the number of χ-intervals *N*_*I*_ and on the CNN parameters (ε, θ). Instead of directly comparing the paths, which is difficult due to changes in the number of clusters and edges as the parameters vary, we examined the spectrum of the infinitesimal generator Q . To this end, we used the Square Root Approximation (SqRA) of the infinitesimal generator,^23,24^ which allows the operator Q to be discretized into a rate matrix **Q**, whose entries *Q*_*ij*_ are proportional to the transition rates between connected clusters and are defined as

26 $$Q_{\mathrm{ij}}\propto \sqrt{\frac{\pi_{j}}{\pi_{i}}}$$

where π_*i*_ and π_*j*_ are the Boltzmann weights of the clusters. Then, we have solved the eigenvalue problem for the rate matrix **Q**, for each system under investigation. The first three eigenvalues κ_1_, κ_2_, κ_3_ show weak sensitivity to the parameters (SI, Figures S5–S7). As *N*_*I*_ increases, with fixed ε and θ, the eigenvalues quickly reach a plateau: for the two-dimensional example, the plateau is already reached with *N*_*I*_ = 3, while for molecular systems, *N*_*I*_ = 5 is sufficient. Varying ε and θ, with fixed *N*_*I*_, reveals that the number of clusters decreases monotonically with ε, and the corresponding eigenvalues shift toward zero until convergence. The parameter θ has only a minor effect on both cluster count and eigenvalue spectrum. Therefore, within wide ranges, the main dynamic characteristics detected by MoKiTo are stable, with *ε* representing the most influential parameter as it controls the granularity of the MKM.

## V Discussion

The dynamics of a molecular system are highly complex and can be represented as a network of pathways connecting clusters of similar configurational states. The idea behind MoKiTo is that molecular dynamics can be decomposed into dynamic processes, known also as Koopman modes, associated with different relaxation time scales. The process associated with the leading nontrivial Koopman eigenfunction ψ_1_ represents long-term transitions, such as protein folding/unfolding or biomolecular binding/unbinding events, and indicates that the molecular system evolves over time along a preferred direction. Then, we use the χ-function, a transformation of ψ_1_ via eq 7, to order the data according to their macroscopic features, facilitating subsequent clustering based on structural similarities.

The first example is particularly useful in showing the power of MoKiTo. The potential energy function has four macro-states and the χ-function reveals correctly the two macroscopic regions and the transition region. Although it would be possible to cluster the initial states *X*_0_ without using the χ-function, the reaction coordinate facilitates the clustering of states with similar macroscopic properties by quantifying how far the intermediate states are away from the starting state (χ-value 0) or close to the end state (χ-value 1).

The other examples show that MoKiTo also applies well to molecular systems of several orders of size. Dichloroisobutene is a small molecule with two macro-states and two possible pathways, the clockwise and counterclockwise rotation of the torsion angle Ψ. MoKiTo captures these properties and reveals that the red configurational states in Figure 4(D), i.e., with both chlorine atoms staggered with the methylene group, are the most stable states of the system. This is the expected result, since strain is minimized by distancing chlorines and the methyl group, which is more sterically demanding than the methylene group due to an additional hydrogen.

The third example shows that MoKiTo can be used to assess the quality of a reaction coordinate. The dynamics of VGVAPG are characterized by the opening and closing of the salt-bridge between the positively charged N-terminus and the negatively charged C-terminus. Intuitively, one might think that the distance between the extreme atoms of the peptide is a sufficient reaction coordinate. Instead, via MoKiTo we revealed more complex dynamics involving two possible rotations of the second residue of the hexapeptide.

Finally, the fourth example shows how to use MoKiTo to identify pathways in the folding/unfolding process of a protein. In contrast to previous examples, where we used conventional MD simulations to sample the representative states of the state space, we used STMD simulations. Indeed, the key requirement for MoKiTo is to have a broad representative set of states regardless of how these were sampled. By contrast, to estimate the χ-function, the short trajectories should respect the true dynamics of the system, but it is not necessary that these trajectories reach a state of thermodynamic equilibrium. We identified two dominant pathways from the unfolded to the folded state. In the pathway highlighted in blue in Figure 6, helix *H*_3_ bends earlier than *H*_1_ and *H*_2_; by contrast, the red pathway exhibits an earlier bend of *H*_1_. These observations are consistent with previous results in ref 55. From Figure 6D, we estimated the difference between the activation free energy barriers of the two pathways to be 4.5 *k*_B_ *T*, in close agreement with the value 4.8 *k*_B_ *T* reported in ref 83. Also, mixed pathways appear from the MKM in Figure 6, where helices form cooperatively at similar time scales, confirming the more recent findings in ref 84.

## VI Conclusion

Traditional tools to pathway analysis such as MSMs and TPT are rigorous and widely used, but they typically require explicit discretization and dimensionality reduction. MoKiTo offers a topology perspective: given an ordering parameter χ representing the leading slow mode, it constructs an MKM and identifies reaction pathways as graph objects, together with energy diagrams to weigh the pathways. We learn χ with ISOKANN, which can be trained on short trajectories that do not reach thermal equilibrium and does not require prior definition of macro-states as discrete state space subsets, eliminating the need for detailed prior knowledge of the system. Across our tests, MoKiTo recovered known mechanisms of villin headpiece subdomain, confirming previous results obtained by other methods.

MoKiTo alone does not provide absolute kinetics (rates, MFPT, flows) and still requires the support of external tools, e.g., SqRA, to calculate relevant kinetic quantities. For this reason, MoKiTo should be considered a complementary tool that elucidates the mechanism and topology of pathways by interacting with established kinetic models.

There is room for improvement for MoKiTo. First, the backward Kolmogorov equation for the χ-function can be turned into a variational principle. This would provide a formulation of the problem that depends only on equilibrium expectations that can be evaluated from independently sampled Boltzmann configurations, including enhanced-sampling or Monte Carlo data, hence trajectory-free. Second, the user parameters in MoKiTo can be chosen more objectively by analyzing the eigenvalue spectrum of the generator on the MKM. For this purpose, we envision automated selection of (*N*_*I*_, ε, θ) based on spectral stability and robustness of the leading eigenvectors. This development would turn MoKiTo into a self-tuning tool providing greater confidence in the recovered pathways.

## Supplementary Material



## Data Availability

The software used for this study is openly available on GitHub at https://github.com/donatiluca/MoKiTo. The complete data set, including original MD trajectories and torch arrays representing pairwise distance matrices, is archived on Zenodo (DOI: 10.5281/zenodo.14229803) and securely stored on the Zuse Institute Berlin server, and can be made available upon reasonable request.

## Notes

Additional information regarding the implementation of the algorithm used to train the FNN within the ISOKANN method; convergence of the χ-function for each example, are available in the SI appendix (Figures S1–S4) (PDF)
