# Supplementary material for: Topological Analysis Reveals Multiple Pathways in Molecular Dynamics
Source: J Chem Theory Comput. 2025 Oct 10;21(20):10385–97. doi: 10.1021/acs.jctc.5c00819 (PMC12573761; doi:10.1021/acs.jctc.5c00819)
Supplement: Supplementary file 1 [file ct5c00819_si_001.pdf]

Supporting Information for:

Topological analysis reveals multiple pathways in molecular dynamics

Luca Donati*,[1,2] Surahit Chewle,[2] Dominik St. Pierre,[3,2] Vijay Natarajan,[4,2] and Marcus Weber[2]

[1] *Freie Universität Berlin, Department of Mathematics and Computer Science, Arnimallee 22, D-14195 Berlin, Germany*

[2] *Zuse Institute Berlin, Takustr. 7, D-14195 Berlin, Germany*

[3] *Freie Universität Berlin, Department of Biology, Chemistry and Pharmacy, Arnimallee 22, D-14195 Berlin, Germany*

[4] *Indian Institute of Science, Department of Computer Science and Automation, 560012 Bangalore, India*

(*Electronic mail: donati@zib.de)

I. Additional description of ISOKANN algorithm and related figures

Figs. S1, S2, S3, S4 show the χ-function (A), the loss functions (B) and the χ-convergence (C), respectively for the two-dimensional system, the 33-Dichloroisobutene molecule, the VGVAPG hexapeptide and the villin headpiece subdomain.

The χ-function was determined by means of the ISOKANN algorithm that iteratively trains a Feedforward Neural Networks (FNNs). The training of the FNN takes place via a nested loop:

- The outer for-loop is the actual modified power method that converges the arbitrary function f_k to the χ-function:

$$\begin{cases} f_{k+1} = S\mathscr{K}_\tau f_k \\ \lim_{k\to\infty} f_{k+1}(x_{0,n}) = \chi . \end{cases} , \tag{S1}$$

  where S is the shift-scale function S as in eq. 16 and $\mathscr{K}_\tau$ is the Koopman operator. We call "iterations" the number of times this loop is applied. In order to verify convergence, we solve the linear regression problem

$$\min_{a_1,a_2} \| f_{k+1} - a_1 f_k - a_2 \| , \tag{S2}$$

  and verify that the slope a_1 is

$$1 - \varepsilon < a_1 < 1 + \varepsilon , \tag{S3}$$

  where ε is a small threshold. Figs. S1-(C), S2-(C), S3-(C), S4-(C) show the value a_1 at each iteration.

- The internal for-loop is the training of the FNN that takes place at each iteration k using techniques like batch processing, early stopping and L^2-regularization to ensure robust performance and to prevent over-fitting. The training loss function is the Mean Squared Error (MSE) between the model's predictions and the actual target values:

$$MSE = \frac{1}{N} \sum_{i=1}^{N} (f_{k_i} - \hat{f}_{ki}) , \tag{S4}$$

  where f_{k_i} are the true values of the function f_k evaluated at iteration k, $\hat{f}_{ki}$ are the corresponding predictions, and N is the number of samples. Before FNN training begins, the

initial dataset, i.e. the dataset of states X_0 representing the state space, is divided into training states (80%) and test states (20%) which are used to estimate the validation loss:

$$MSE^{\text{val}} = \frac{1}{N}\sum_{i=1}^{N}(f_{k_i}^{\text{val}} - f_{k_i}), \tag{S5}$$

We call "epochs" the number of times this loop is applied.

The Python code of the ISOKANN implementation used is available at `https://github.com/donatiluca/MoKiTo`.

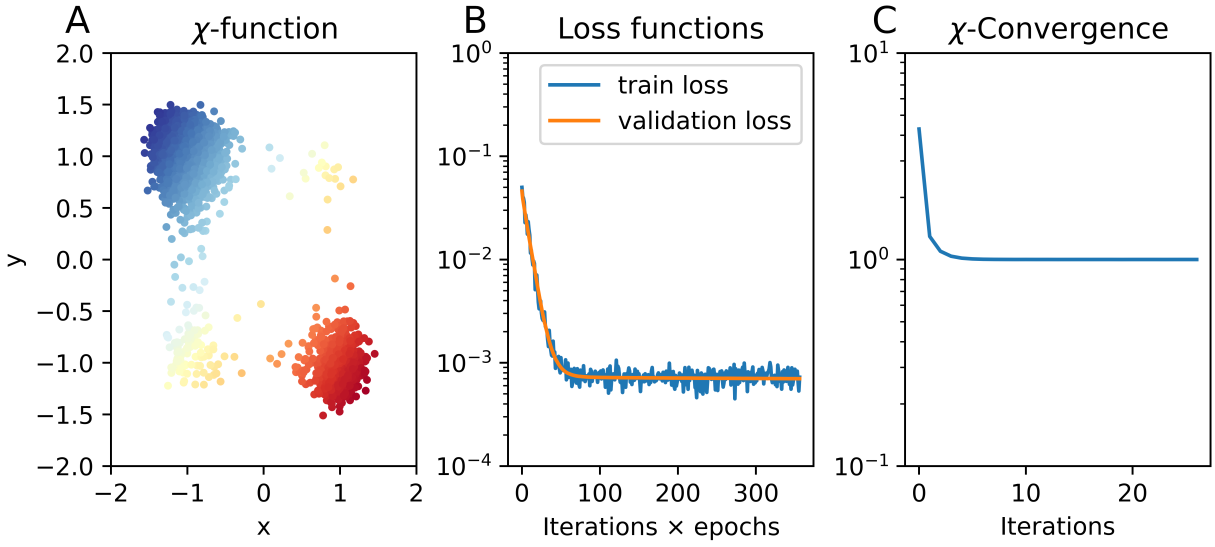

FIG. S1. (A) χ-function of the two-dimensional system projected onto the Cartesian coordinates x and y; (B) Validation and training loss functions; (C) χ-convergence.

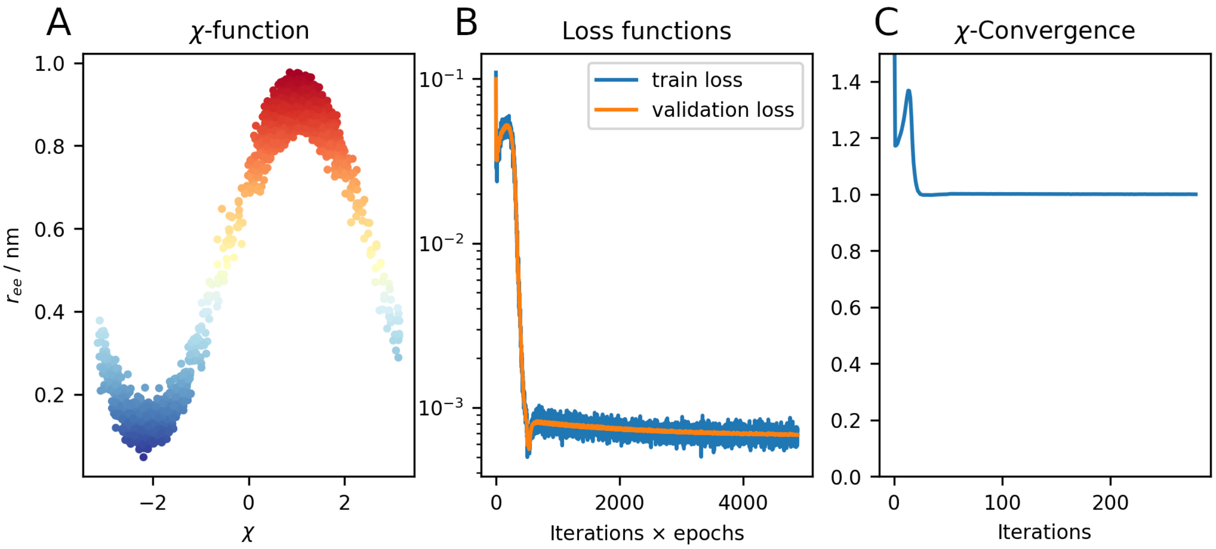

FIG. S2. (A) χ-function of 33-Dichloroisobutene molecule projected onto the torsion angle Ψ; (B) Validation and training loss functions; (C) χ-convergence.

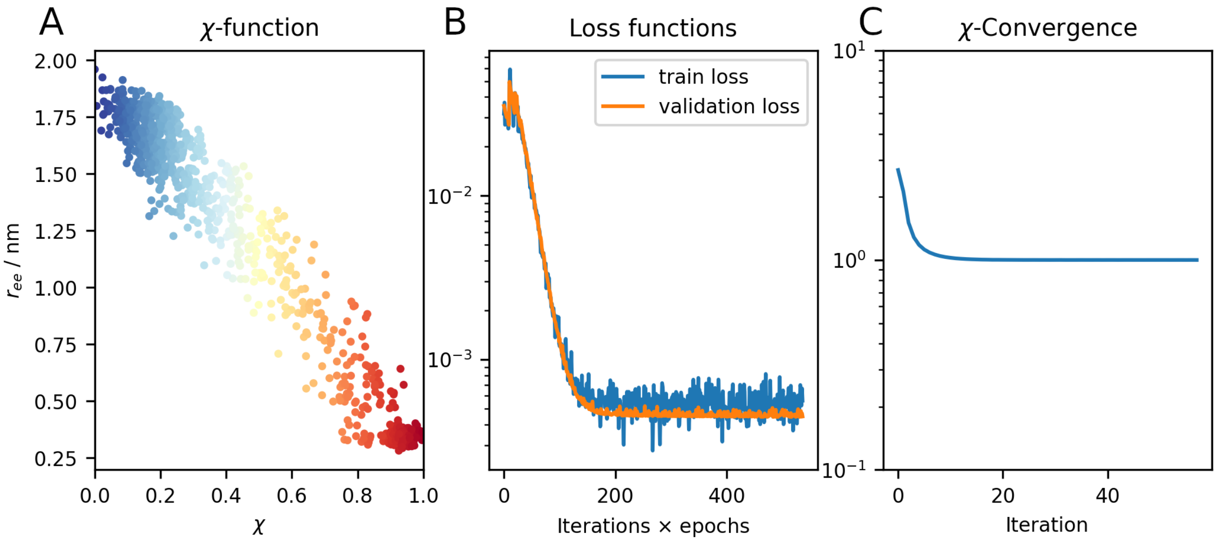

FIG. S3. (A) χ-function of hexapeptide VGVAPG projected onto the end-to-end distance r_{rr}; (B) Validation and training loss functions; (C) χ-convergence.

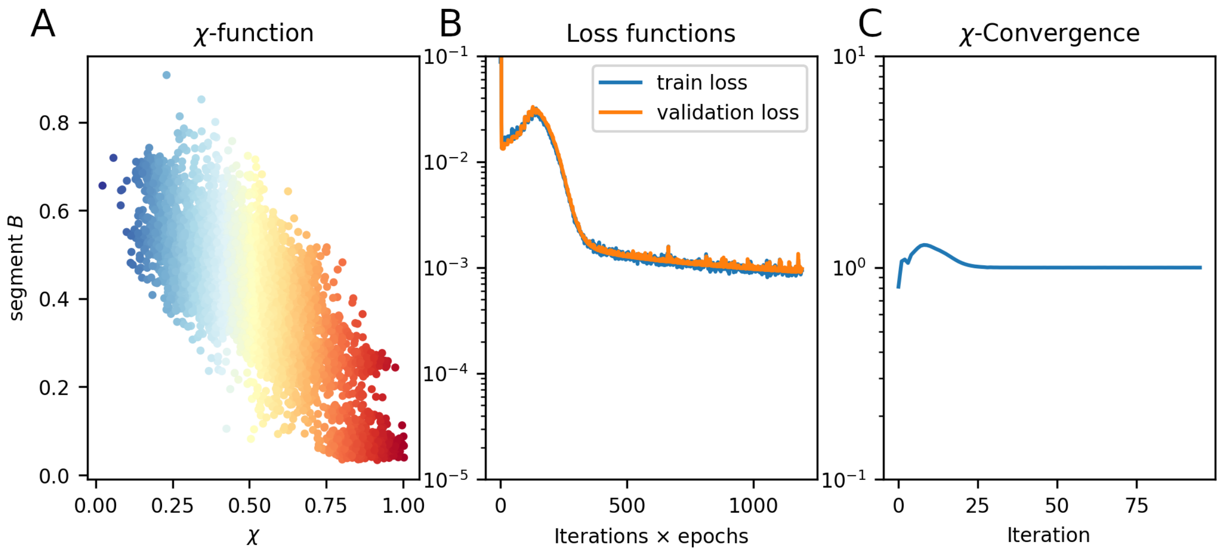

FIG. S4. (A) χ-function of villin projected onto the RMSD of the segment B; (B) Validation and training loss functions; (C) χ-convergence.

II. Sensitivity analysis to χ-discretization and CNN parameters

To quantify the robustness of MoKiTo, we measured how the eigenvalue spectrum of the infinitesimal generator varies with the number of χ-bins N_I used to order states and the CNN parameters (ε, θ) used to cluster states within each bin. Fig. S5 shows the dependence of the first three non-trivial eigenvalues κ_1, κ_2 and κ_3 of $\mathbf{Q}$ on the number of χ-bins N_I for all systems (panels A–D). Fig. S6 reports, for the two-dimensional system (A) and 33-Dichloroisobutene (B), how the number of clusters (left panel) and the leading eigenvalues (right panel) vary with the CNN parameters (ε, θ) at fixed N_I. Fig. S7 mirrors Fig. S6 for VGVAPG (A) and the villin headpiece (B).

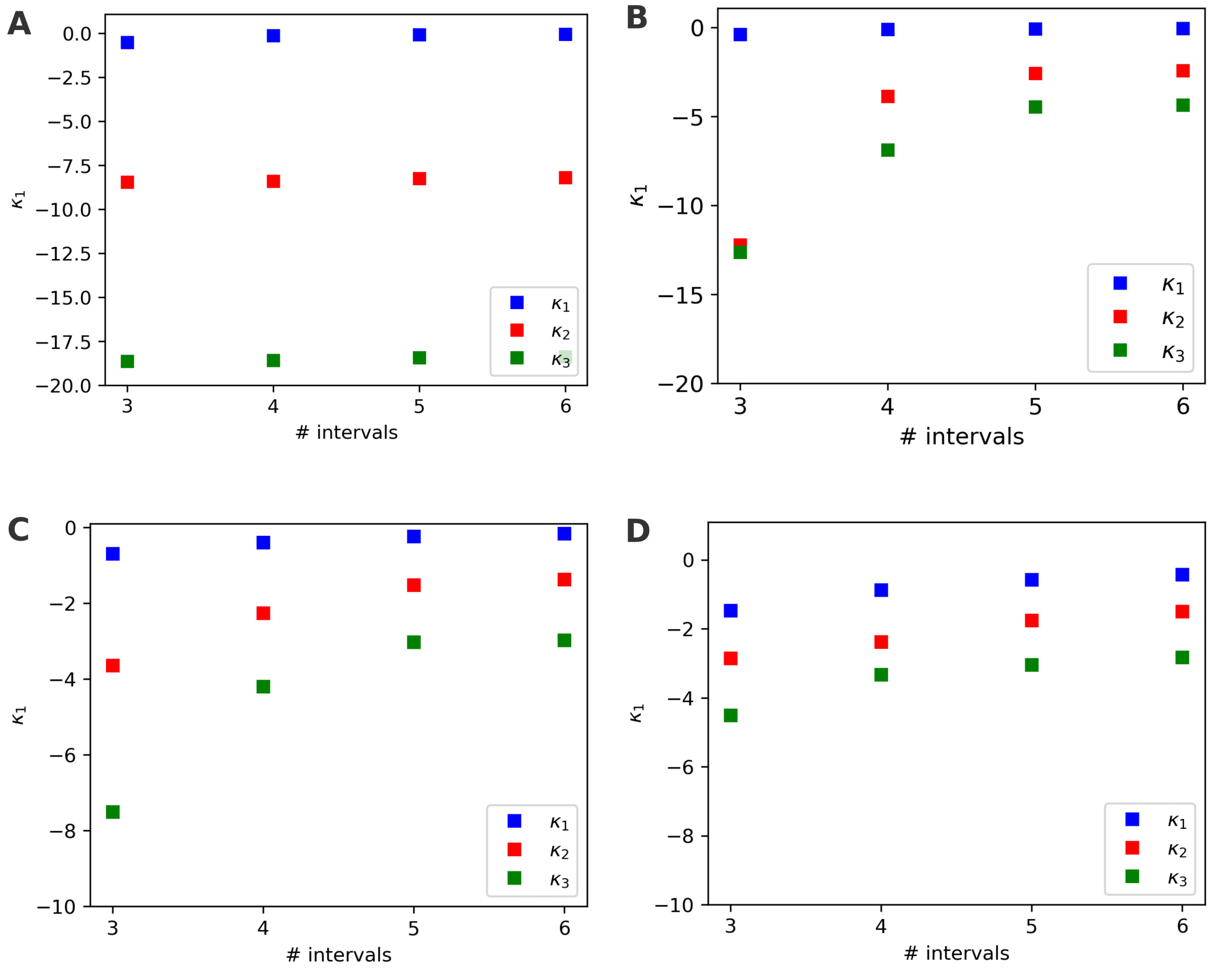

FIG. S5. Dependence of eigenvalues on the number of intervals used to discretize the χ-function. (A) Two-dimensional system; (B) 33-Dichloroisobutene molecule; (C) VGVAPG hexapeptide; (D) Villin headpiece subdomain.

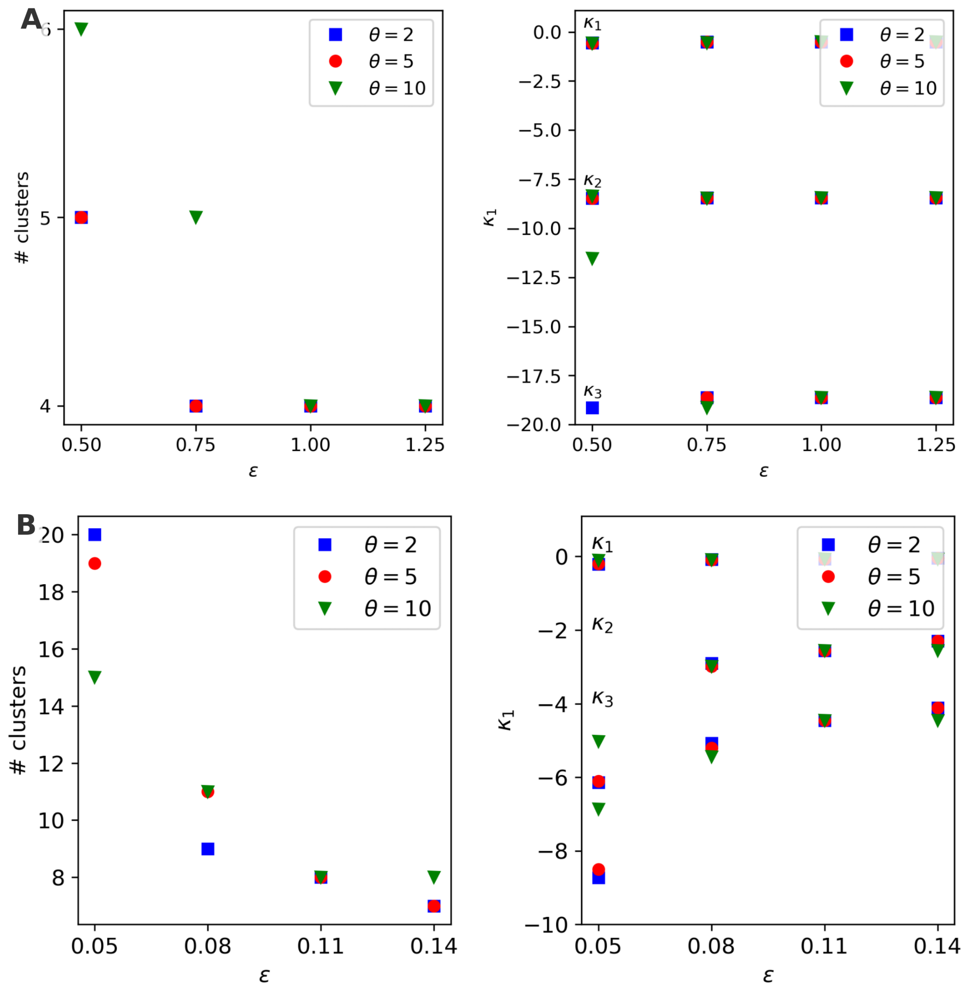

FIG. S6. Dependence of number of clusters (left) and eigenvalues (right) on the parameters ε and θ. (A) Two-dimensional system; (B) 33-Dichloroisobutene molecule.

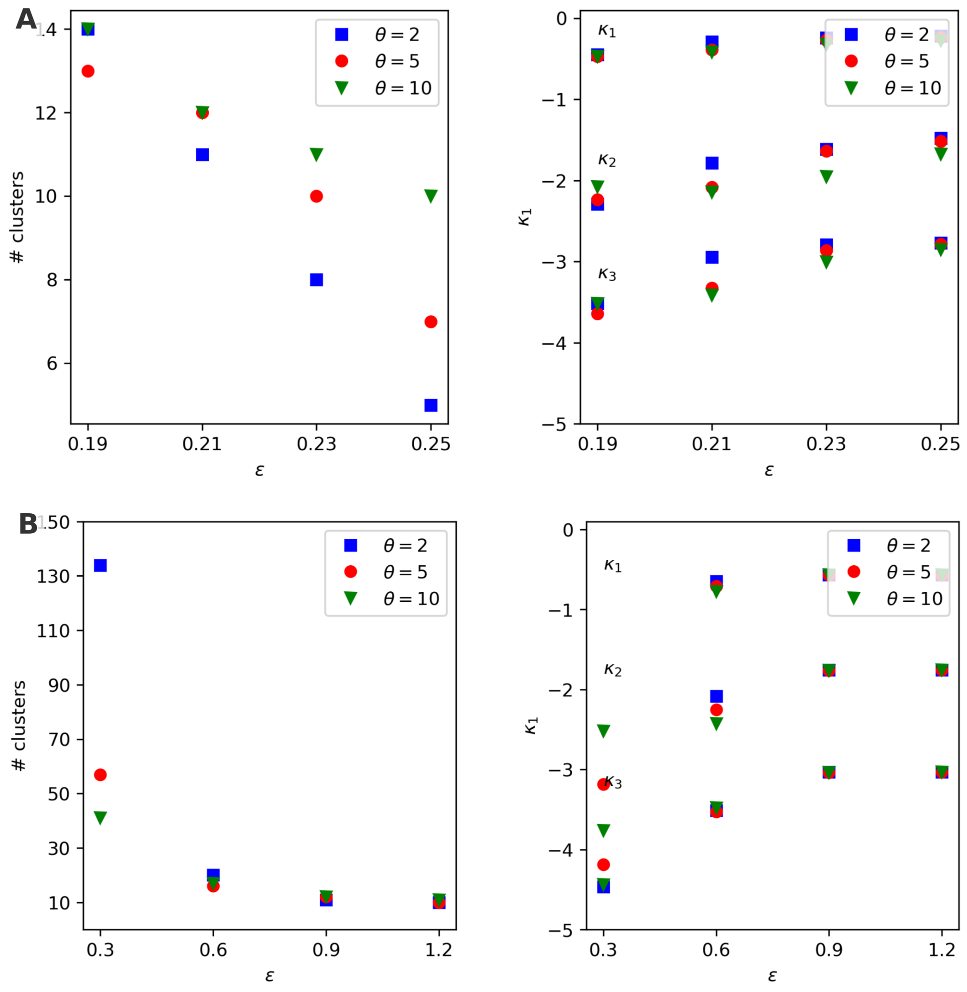

FIG. S7. Dependence of number of clusters (left) and eigenvalues (right) on the parameters ε and θ. (A) VGVAPG hexapeptide; (B) Villin headpiece subdomain.
